# The Role of Acculturative Stress on the Mental Health of Immigrant Youth: A Scoping Literature Review

**DOI:** 10.1007/s10597-024-01351-x

**Published:** 2024-09-06

**Authors:** Doukessa Lerias, Tahereh Ziaian, Emily Miller, Nancy Arthur, Martha Augoustinos, Tara Pir

**Affiliations:** 1 University of South Australia (Justice and Society), Adelaide, South Australia Australia; 2 University of South Australia (UniSA Business), Adelaide, South Australia Australia; 3 University of Adelaide (Psychology), Adelaide, South Australia Australia; 4 Institute for Multicultural Counseling and Education Services, Los Angeles, California USA

**Keywords:** Acculturation, Acculturative stress, Immigration, Youth mental health, Late adolescence, Emerging adulthood

## Abstract

Acculturation after settlement has been identified as a risk factor affecting the mental health of immigrant youth. Increasing rates of immigration and expanding populations of immigrant youth mean that addressing their mental is a priority. Acculturative stress is the stress-response resulting from the effects of multiple stressors that result from the need to acculturate. Among youth within the developmental stages of late adolescence and emerging adulthood, increased sensitivity to stress, and developmental demands, impact their mental health. The effects of acculturative stress place an additional burden on the mental health of immigrant youth. This scoping review examined existing literature that investigated a variety of relationships between acculturative stress and youth mental health. A comprehensive search strategy that focused on studies involving youth, mainly aged between 15-24, with a proximal history of international migration, published between 2012-2022, resulted in a collection of fifty-three studies. This review examined significant relationships between acculturative stress and major depression, anxiety disorders, eating disorders, substance misuse, behavioural problems and poor psychological wellbeing. This scoping review was truly explorative as it included youth from immigrant minorities, had no geographical limits, and included various study designs. Acculturative stress continues to be an important contributor to the mental health of youth who have a proximal history of international migration. This review provided an exploration of the state of research, identified the importance of the settlement context, and provided recommendations for the direction of future studies, supportive policies, and practice considerations, related to the mental health of immigrant youth.

There are currently 281 million people who are immigrants^1^ globally, and this has increased since the year 2000, by 131 million people (International Organization for Migration [IOM] 2024; McAuliffe & Oucho, 2024). Children under the age of 18 make up 13%, and youth aged between 15–24 make up 11% of the total group of immigrants globally (IOM 2024). Between 1990 and 2020, numbers of immigrant children under 18 years of age increased by approximately 50%, and numbers of immigrant youth aged 15-24 increased by 43% (IOM 2024; McAuliffe & Oucho, 2024). The pathways to international migration are incredibly diverse and can be complex, as immigration policies and the context of reception for immigrants, continuously changes (Hodes et al., 2018; McAuliffe & Oucho, 2024). There is also growing inequality in pathways to immigrating that can affect the safety and welfare of immigrants (McAuliffe & Oucho, 2024). Major regions that host immigrants include North America, the Arab States and Europe, and in many countries in these regions, immigration has become the main source of population growth (McAuliffe & Oucho, 2024). Even after settlement has been achieved, it is common for children who have been born in some settlement countries to be considered immigrants as well (Batalova, 2024; McAuliffe & Oucho, 2024).

Approximately 45 million immigrants have been forcibly displaced, with 35 million being registered with United Nations’ agencies as refugees (IOM 2024; McAuliffe 2024). Among refugee groups, 41% are under the age of 18, and there has been a 230% increase in in this group since 1990 (IOM 2024; McAuliffe 2024). Countries such as Canada, Australia and the United States have markedly increased the number of refugees that they have welcomed for resettlement, however, global conflict is at a historical high and the prospect of peace at a historical low (McAuliffe & Oucho, 2024). There is an urgent need for the safe and effective long-term resettlement of refugees globally (Birch, 2023; Hodes et al., 2018).

Being of immigrant background has been identified as a risk factor for serious mental health problems that can emerge in late adolescence, in countries with high immigration (Kim et al., 2019; McMahon et al., 2017). Acculturation is the psychological and behavioural adaptation to a new and dominant culture and requires substantial emotional, cognitive and social resources (Berry et al., 1987). Acculturation to a settlement country is complex and demanding, and may require a vast array of accomplishments, that can include new communication and social skills, learning styles and the adoption of new cultural values (Berry et al., 1987). However, over time and development across the life span, acculturation requires deeper transformation of identity and the synthesis of both heritage and settlement histories into a bicultural identity and way of life (Liebkind et al. in Sam & Berry, 2016). Acculturation has been found to be a risk factor for youth-suicide globally, and acculturating refugee and migrant youth are both at risk of self-harm due to poor mental health (Abraham & Sher, 2021). Other common mental health problems related to the settlement experiences and acculturation of immigrant youth include; depression, anxiety, eating disorders, substance misuse and behavioural problems (Abdulhamed et al., 2022; Blackmore et al., 2020; Hilario et al., 2015; Kim et al., 2019). Therefore, the mental health and wellbeing of immigrant youth becomes a priority in countries that accept immigrants, and understanding the psychological impact of acculturation experiences, could be key in preventing or treating serious mental health problems among growing populations of immigrant youth.

Berry and Annis (1974) originally used the term *‘acculturative stress’* to describe the negative emotional states that included reduced physical health, symptoms of anxiety, depression, grief and loss among migrant, refugee and Indigenous minority groups, brought on specifically by stressors related to acculturation and assimilation (Berry & Annis, 1974; Berry et al., 1987). According to Berry and Annis (1974), acculturative stress was not ‘culture-shock’, but a pervasive, negative stress response resulting from the expectations of assimilation, which included the cessation of one cultural way of life, and the introduction of another, that could explain the variability in the mental and physical health outcomes of immigrants and minority groups in countries such as Canada (Berry & Annis, 1974; Berry et al., 1987; Williams & Berry, 1991). In recent years, as attitudes and policies related to minority groups have evolved in countries that accept immigrants, acculturation is currently understood as the bicultural integration of one’s heritage culture and identity into their settlement country, culture and context (Sam & Berry, 2016). The term ‘*acculturative stress*’ has also followed, and refers to the negative emotional and physical experience in response to the complexity of acculturating, and the accumulation of many acculturation challenges (Sam & Berry, 2016). Acculturative stress related to immigration, is not the same as general life stress, but a negative psychological experience directly resulting from managing the ongoing demands of a bicultural environment, bicultural group membership and bicultural identity throughout one’s lifespan (Castillo et al., 2015a, 2015b; d’Abreu et al., 2019; Rodriguez et al., 2002).

Late adolescence is a critical developmental period where sensitivity to stress and negative emotional states is heightened, executive functioning is forming, and relationships and identity take focus (National Academies of Sciences Engineering Medicine [NASEM] 2019). In many settlement countries, the end of adolescence is followed by emerging adulthood (Arnett, 2024; Dimitrova, 2017). Emerging adulthood is a developmental trend that although was originally identified in the United States, has been observed among youth aged 18 and up to 29 years, across Asia, Africa, Europe and North and South America, Australia, and New Zealand, and among minority groups in multicultural countries (Arnett, 2024; Dimitrova, 2017; Syed & Mitchell, 2013). It is a period defined as the preparation for adult responsibilities that focuses on relationships, identity, managing responsibilities and academic and vocational performance (Arnett, 2024; Dimitrova, 2017). Adolescents and emerging adults face frequent and significant transitions and experience an intense adaptive process that requires critical interpersonal and environmental resources (Arnett, 2024; Dimitrova, 2017; NASEM 2019). This is a time when learning and adaptation is heightened but so is the risk of emerging mental health problems (Arnett, 2024; Dimitrova, 2017; Syed & Mitchell, 2013). For youth with a proximal history of international migration that are facing these transitions, and accumulation of acculturative stressors, limited protective resources, and the experience of acculturative stress, can cause substantial obstacles in achieving these transitions, and consume psychological resources. Given the vulnerabilities associated with developing youth, acculturative stress could be a significant contributor to the mental health problems in immigrant youth who face the complex acculturative process of developing into bicultural adults in their settlement country.

The purpose of this scoping literature review was to explore the recent empirical evidence on the relationship between acculturative stress and known mental health problems among youth predominantly aged between 15-24, with a proximal history of international migration, who are facing long-term acculturation to their settlement country. In the context of recent global, immigration trends and settlement contexts, this review aimed to explore whether acculturative stress is a relevant and important aspect to address in future research efforts related to immigrant youth, and whether it should be addressed when supporting the mental health and wellbeing of youth.

Therefore, scoping literature review methodology was used to answer the question: *In what ways does acculturative stress impact the mental health of immigrant youth?* This review had the following aims:

1. To explore whether acculturative stress plays a role in the mental health of immigrant youth by reviewing recent research studies focused on this topic.
2. To understand and report the nature of this relationship by presenting the findings of included studies in a manner that may be considered in supportive practice and policy making related to the mental health and wellbeing of immigrant youth across a variety of settlement countries.
3. To provide recommendations for the direction of future research related to the mental health and wellbeing of immigrant youth.

## Method

This scoping review was conducted with guidance from the Joanna Briggs Institute (JBI) for scoping reviews and the Preferred Reporting Items for Systematic Reviews and Meta-Analyses Scoping review extension (PRISMA-ScR) reporting guidelines (Page et al., 2021; Peters et al., 2017; Tricco et al., 2018).

### Eligibility criteria

#### Population

The United Nations defines youth as being between the ages of 15 to 24 (UNDESA 2013). This review only included studies of reference groups where the majority of the sample reflected ages included in this age range. This included studies that focused on late adolescence (15-18 years) and the early stages of emerging adulthood (15-24years). This review included search terms such as; high school, college, university, students, youth, young adults, and emerging adults and included studies if the mean, mode or majority age were between 15–24 years. This review attempted to avoid studies that focused on childhood and early adolescent development by excluding studies where the mean, mode and majority age was less that 15 years of age.

#### Concept

This review focused on ‘acculturative stress’, otherwise referred to as ‘acculturation stress’ (Sam & Berry, 2016). Studies referring to ‘acculturative stress’ or ‘acculturation’ and ‘stress’ as a keyword or key theme were included in the review. This review included studies whose primary aims contained the evaluation of acculturative stress and mental health problems such as; depression, anxiety, suicide, body image disturbance and eating disorders, somatisation, substance misuse, behavioural problems such as delinquency, risk-taking and impulse control problems; as well as poor psychological wellbeing, for example reports of identity distress, Covid-anxiety, self-esteem, or psychological distress.

#### Context

This review included studies that sampled youth who had a personal or proximal family history of international migration. This included reference groups such as immigrant, migrant, refugee, immigrant minority, newcomer, first-generation immigrant, and second-generation immigrant. This review focused on the post-settlement or post-migration experiences of youth who were permanent residents of their settlement country. Studies referring to acculturative stress in temporary residents were excluded as they were not in the post-migration period (Kirmayer et al., 2011; Nickerson et al., 2019).

#### Sources

This review only included primary, empirical studies that used quantitative, qualitative, mixed methods or experimental designs. Peer reviewed studies, and grey literature such as commissioned studies, conference abstracts with reports, and dissertations were included. Only studies published between 2012 to 2022 were included. Studies were accepted from all countries and locations. Only reports available in English were able to be reviewed.

#### Search Strategy

A 2-step database search strategy was conducted across several databases including PsycInfo, SCOPUS, Medline, PsycExtra and Social Science Database. An example of the search strategy employed to find studies is presented in Table 1. In addition, TROVE, APO, Google and Google scholar were searched with key terms pertaining to youth and acculturative stress. Reviewers also conducted a citation search of relevant articles.

**Table 1:** Example of search strategy used to find studies on OVID databases such as Medline and PsycInfo Ovid databases such as Medline and PsycInfo (ti) title, (ab) abstract, (kf) keyword heading

Participants AND		Concept AND	Context
1.	Youth OR Student* School leavers Adolescent* Emerging adult* Young adult*	Acculturative stress OR Acculturation stress OR Acculturat* AND stress	
2.	Youth OR Student* School leavers Adolescent* Emerging adult* Young adult*	Acculturative stress OR Acculturation stress OR Acculturat* AND stress	Minorit* Refugee* Migrant* Immigrant* Newcomer

Similar search strategies were used on other databases such as SCOPUS

### Study Selection and Data Extraction

**Fig. 1:**
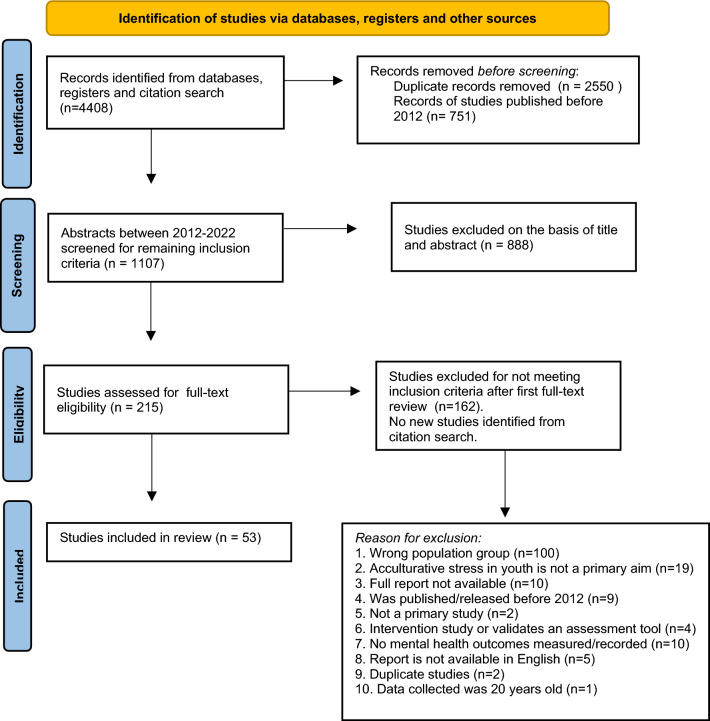
Study screening and selection process using the Preferred Reporting Items for Systematic and Meta-Analyses (PRISMA-2020) reporting methodology (Page et al., 2021)

Endnote 20^TM^ and Covidence systematic review software were used to conduct the review (The Endnote Team, 2013; Veritas Health Innovation, 2022). Duplicates were removed followed by references published before 2012. Following a pilot test and finalization of the inclusion criteria, independent screens of the title and abstract were conducted by the first and third author. Interrater agreement for the abstract screening phase was 89.2 % (Cohens Kappa 0.68). After inclusion criteria were finalized, included studies were subjected to an independent, full text review by the first and third authors. The level of agreement was 93% (Cohens Kappa: 0.83). Abstract and full-text screening conflicts were resolved with discussion and the second author was available to assist with consensus. A customised data extraction tool was developed, and extraction of relevant data from each report was conducted using Covidence systematic review software and completed by the primary author (Veritas Health Innovation, 2022). Figure 1 further illustrates the screening and selection process.

## Results

Fifty-three studies satisfied inclusion criteria and their findings were evaluated for their ability to identify a relationship between acculturative stress and mental health problems in groups representing immigrant youth. This included studies that investigated youth that identified as having a proximal history of international migration, regardless of their race or ethnic background and, studies that focused on groups of youth that identified as belonging to an immigrant minority community within their settlement country. The studies included in this review are listed in Table 2 along with the study location, sample characteristics, mental health outcomes and method of data analysis. This review sought to understand the specific relationship between acculturative stress and common mental health problems among immigrant youth, by identifying significant relationships between these two constructs and any mediators or moderators that may influence this relationship. Following Table 2 is a description of the existing evidence that identifies that acculturative stress plays a role in a range of mental health problems found in youth, and the factors that affect this relationship.

**Table 2:** The location, design, sample and evaluation characteristics of the fifty-three studies included in the scoping review

Reference; Year; Report type.	Settlement country	Study design	Sample description	Mental Health Measure	Mental Health Outcome(s)	Acculturative Stress Measure	Acculturative Stress and Mental Health – Analysis
Alamilla et al. (2020); Peer Reviewed published article	United States	Quantitative	N=3362 non-white students from 30 universities. N= 863 1st & 2nd generation immigrants. Mean age: 20 (SD: 2.1)	Alcohol Use Disorders Identification Test (AUDIT) plus "How often do you drink alcohol before going to a party, club, or other social setting."	Alcohol Misuse	The Pressure to Acculturate (PRESSAC) and Pressure against Acculturation (PRESSACA) subscales from the Multidimensional Acculturative Stress Inventory (MASI); Perceived Discrimination (PD) subscale from the Scale of Ethnic Experience.	Descriptive intercorrelations; Stratified regression modelling; Independent sample t-tests; Blinder-Oaxaca and stratified regression analyses.
Alemi et al. (2021); Peer Reviewed published article	United States	Qualitative	N = 2 mental health clinicians working with refugees, 1 Somali Imam, 4 informal community leaders, 4 Somali youth for a focus group discussion and 21 Somali youth for interviews. Of the youth, 72% refugees. 28% (n=4) sponsored or US born. Age range 18-34, 72% (18) were between 18-25.	Focus group discussions and interviews	Posttraumatic Stress Disorder (PTSD); Psychological distress	Focus group discussions; Interviews.	Conventional content analysis.
Assar (2015); Dissertation	United States	Quantitative	N= 152 college students, with at least one parent of middle-eastern descent. Mean Age: 20.14 (SD: 2.43), Age range: 18-30 with most subjects under 24. 1st & 2nd generation immigrants.	Beck Youth Inventories; Rosenberg Self-esteem Scale	Anxiety; Depression; Self esteem	Social, Attitudinal, Familial, and Environmental Acculturative Stress Scale (SAFE).	Exploratory MANOVA; Pearsons correlations; multiple regression; Stepwise linear regression; Moderated mediation modelling.
Badiee and Andrade (2019); Peer Reviewed published article	United States	Quantitative	N=403 Hispanic college students, 91% of Mexican background. Mean Age: 22.13 (SD: 4.16). 14% 1st generation and 72% 2nd generation immigrants.	Center for Epidemiologic Studies Depression Scale–Revised version (CESD-R); Generalized Anxiety Disorder–7 Scale	Depression; Anxiety	The National Latino & Asian-American Study (NLAAS) - Acculturative Stress Scale, Discrimination Scale, Foreigner Objectification Scale.	Hierarchical multiple regression analysis.
Bhowmik (2021); Peer Reviewed published article	Hong-Kong/ China	Qualitative	N= 20 immigrants of Pakistani, Indian, Filipino, Bangladeshi, Nepalese and mixed Filipino / Nepalese ethnicity. Mixed high school, young professionals and University students. Longstanding residents of Hong-Kong.	Questioning on psychosocial wellbeing	Psychological wellbeing	Participants provided details of their experiences of stress from language and communication barriers, racism, family-related conflicts & small housing.	An ecological approach. Categories and subcategories were informed by methods from Strauss & Corbin (1998).
Cano et al. (2014); Peer Reviewed published article	United States	Quantitative	N=155 Mexican college students. 26.5 % were 1st generation, 48.4 % were second generation immigrants. Mean Age: 22.64 (SD: 6.44)	Center for Epidemiological Studies Depression Scale (CES-D)	Depression	Social, Attitudinal, Familial, and Environmental Acculturation Stress Scale (SAFE).	Bivariate correlations; Path analysis using Chi square test of model fit.
Castillo et al., (2015a, 2015b); Peer Reviewed published article	United States	Quantitative	N=1004 (Table 2, p. 47), Hispanic college students. n=773 were 1st and 2nd generation immigrants. Mean Age: 20.3 (SD: 3.37).	The Center for Epidemiologic Studies Depression Scale (CES-D)	Depression	The Multidimensional Acculturative Stress Inventory (MASI).	Multiple group analysis; Path analysis using Chi square test of model fit.
Chen (2017); Dissertation	United States	Quantitative	N= 175 Chinese / Taiwanese college students 100% 1^st^ and 2nd generation immigrants. Age range: 18-39, Mean Age: 23.64 (SD: 3.58).	The Center for Epidemiologic Studies Depression Scale (CES-D); State & Trait Anxiety Inventory (STAI); Ryff’s Scale for Psychological Wellbeing,	Depression; Anxiety; Psychological wellbeing	Social, Attitudinal, Familial, and Environmental Acculturative Stress Scale (SAFE).	Bivariate correlations; Hierarchical regression analysis; Moderated mediation modelling.
Cheng (2022); Peer Reviewed published article	United States	Longitudinal	N= 386 at time 1 and N=173 at time 2. Hispanic college students. 11% 1st generation and 39.9% 2nd generation immigrants. Mean Age: 23.05 (SD: 7.43) at time 1.	Patient Health Questionnaire-9 (PHQ-9)	Depression	Riverside Acculturation Stress Inventory (RASI).	Cross-lagged structural equation modelling (SEM).
Claudat (2015); Dissertation	United States	Quantitative	N=279 Latina women; 1st & 2nd generation immigrants. Age range: 18-24.	The Surveillance subscale of The Objectified Body Consciousness Scale (OBCS); The Objectified Body Consciousness Scale (OBCS); The Social Appearance Anxiety Scale (SAAS); The Eating Attitudes Test-26 (EAT-26); Thin Internalization: Thin/Low Body Fat subscale of the SATAQ-4.	Body Image disturbance and eating pathology	The Societal, Attitudinal, Familial, and Environmental Acculturative Stress Scale - 24 items (SAFE-24).	SEM path analysis; Mediation analysis; Hierarchical regression analysis.
Claudat et al. (2016); Peer Reviewed published article	United States	Quantitative	N= 638 1^st^ and 2^nd^ generation immigrant women. Self-identified as Asian (n = 340; 53.3%) or Latina (n = 298; 46.7%). Mean Age: 19.88 (SD: 3.67).	The Rosenberg Self-Esteem Scale (RSE); The Eating Disorder Examination Questionnaire (EDE-Q); Body Mass Index (BMI).	Eating pathology; Self-esteem.	The Societal, Attitudinal, Familial and Environmental Acculturative Stress Scale - 24 items (SAFE-24).	Bivariate correlations; Independent sample t-tests; Hierarchical moderated regression; Multi group SEM; latent variable SEM.
Corona et al. (2017); Peer Reviewed published article	United States	Quantitative	N=198 LatinX college students Age range: 18 -25; 91% were 1st and 2nd generation Immigrants. Mean Age: 20.6 (SD: 1.78)	DASS21	Depression; anxiety; stress.	Riverside Acculturative Stress Inventory (RASI).	Pearson & point-biserial correlations; Multiple regression analyses; Hierarchical linear regression modelling.
Ehlers et al. (2016); Peer Reviewed published article	United States	Quantitative	N=614, Mexican immigrants. Mean age: 23.5 (S.E.: 0.3), Age range: 18-30. Majority of the sample was 23-24 years. 72% 1st & 2nd generation immigrants.	Face to Face interview with the Semi- Structured Assessment for the Genetics of Alcoholism.	Substance misuse; depression; anxiety, PTSD	The Multidimensional Acculturative Stress Inventory (MASI).	Univariate analysis with two-tailed significance; Chi square analysis; ANOVA; Logistic regression.
Gersick (2015); Dissertation	United States	Quantitative	N=119 Asian youth. Mean Age: 19.42 (SD: 2.19), Age range 18-27. 42.9% 1st and 57% 2nd generation immigrants.	The Global Severity Index (GSI) of the Brief Symptom Inventory (BSI)	Somatization; OCD; depression; anxiety; psychosis & general psychological distress.	Social, Attitudinal, Familial, and Environmental Acculturative Stress Scale – Short form (SAFE).	Pearsons and point-biserial correlations; Hierarchical logistic regression; Hierarchical linear regression.
Goforth et al. (2014); Peer Reviewed published article	United States	Quantitative	N=128 Muslim Arabic speaking adolescents, between the ages of 11 and 21, Mean Age: 15.5 (SD: 2.8). 77% 1st generation, 23% 2nd generation immigrant.	An English and Arabic version of the Youth Self-Report (YSR)	Children's social and emotional functioning; Social competence and total internalizing and externalising problems.	Social, Attitudinal, Familial, and Environmental Acculturative Stress Scale - Children’s Version (SAFE-C).	Zero-order correlations; Simultaneous regression analysis.
Hale and Kuperminc (2021); Peer Reviewed published article	United States	Quantitative	N=129 Hispanic high school students. 72% were first- generation and 28% were 2nd generation. Mean Age: 16.83	Weinberger Adjustment Inventory	Depression; Anxiety; Self-esteem; wellbeing.	The Societal, Attitudinal, Familial, and Environmental Acculturative Stress Scale - 28 items (SAFE-28).	Zero-order correlations; Hierarchical regression analysis; Simple slopes analysis
Hernandez Dubon et al. (2022); Peer Reviewed published article	United States	Quantitative	N= 224 Latinx youth. 28%Born Overseas versus 72% local born youth.82% of mothers and 80% of fathers were born overseas. Mean age: 20.97 (SD: 1.69)	CORE Alcohol and Drug Survey (CADS)	Regular tobacco use	Social, Attitudinal, Familial, and Environmental scale- 24 items (SAFE-24).	Confirmatory factor analysis; Bivariate correlations; Mediation analysis.
Ibrahim-Joudeh (2014); Dissertation	United States	Quantitative	N=80 High school students and N=85 Parents. 16% of parents were born in the USA. 74% born overseas making 74% of the sample at least 2nd generation immigrants. Age range 14-18, mean age 15.9 (SD: 1.12) 91.4% of the sample is 15+ years old.	DASS21 Rosenberg Self-Esteem Scale.	Psychological adjustment measured by depression, anxiety and general stress; Used a total score of the DASS21 to assess general psychological distress; Self-esteem.	Social, Attitudinal, Familial, and Environmental scale – Children’s version (SAFE-C).	Independent t-tests; Pearson correlations; Paired sample t-tests.
Jankowski et al. (2020); Peer Reviewed published article	United States	Quantitative	N=1,072 Hispanic and Asian college students. Mean age: 19.7 (SD: 1.62). 67.1% U.S. born; 33.9% born overseas.	15-item Brief Comprehensive Effects of Alcohol Scale; Alcohol Use Disorders Identification Test (AUDIT)	Alcohol misuse; alcohol expectancies.	Multidimensional Acculturative Stress Inventory (MASI) modified for Spanish language references to all country-of-origin languages.	Moderated mediation model; Complex sample modelling.
Jeon (2022); Peer Reviewed published article	South Korea	Quantitative	N=1207 2nd generation immigrants with at least 1 parent born outside of South Korea. Mean age: 14.96 (SD = .350), Age range:14–17.	The self-esteem variable comprised 4 items including questions: ‘I feel proud of myself’ and ‘I have expectations of being a good person’, as developed by Park & Oh (1992); 9 items measuring career barriers, such as ‘self-understanding’, ‘lack of career and occupational information’, and ‘eco- nomic difficulties’.	Self Esteem; Cognitive appraisals of career barriers.	10 items, such as ‘stress of poor spoken Korean’ and ‘discrimination for having foreign parents’, which were developed by Hong (2004).	Bivariate correlations; Hierarchical regression analysis; Simple slope test.
Katsiaficas et al. (2013); Peer Reviewed published article	United States	Longitudinal	N=304 1st generation (48%) and 2nd generation (52%). Mean Age: 15.7 (SD: 0.83).	12-item anxiety-depression component from the internalizing scale of the Youth Self Report (YSR)	Anxiety; Depression.	Social, Attitudinal, Familial, and Environmental Acculturative Stress Scale - Short Form (SAFE-Short).	Bivariate correlations; Independent sample t-test; Mediation modelling; Moderated mediation modelling;
Kim et al. (2015); Peer Reviewed published article	South Korea	Quantitative	N=144 North Korean refugee youth. Mean age: 18.2 (SD: 2.03). Age range: 13-21.	The Hopkins Symptoms Checklist (HSCL-25); UCLA Posttraumatic Stress Disorder Index (DSMIV) Adolescent Index; Ego Resiliency Scale	Anxiety; Depression: PTSD.	Acculturation Stress scale (ASC).	Confirmatory factor analysis; Multi factor structural equation modelling.
Lee et al. (2018); Peer Reviewed published article	United States	Longitudinal	N= 477 Mexican female college students n=234 in Michigan and n=243 in Arizona. 1st & 2nd generation immigrants. Mean age: 19.8 (SD: 2.4)	Ecological momentary assessment (EMA); Drinking Schema true/false questionnaire.	Alcohol Misuse & Drinking identity.	Social, Attitudinal, Familial, and Environmental Acculturative Stress scale – 26 items (SAFE-26).	Correlations; Stepwise multivariate modelling.
Leffler (2015); Dissertation	United States	Quantitative	N=195 LatinX students. Age range:18-25, with the majority between 18-21. 80.5% were of Mexican Heritage, 4.1% were 1st and 67.2% were 2nd generation immigrants.	Centre for Epidemiologic Studies Depression scale (CES-D); The 20-item Zung Self-Rating Anxiety Scale	Depression; Anxiety.	Autonomy Conflicts subscale of the Acculturation Gap Conflicts Inventory (AGCI); The Autonomy Conflicts subscale of the AGCI was modified to measure school-based acculturative stress.	Pearsons correlations; Multiple regression analysis.
Maiya et al. (2021); Peer Reviewed published article	United States	Quantitative	N=1410 LatinX college students. Mean Age: 19.71(SD: 1.7), Age range: 18-25. 1st and 2nd generation immigrants. 66.5% of mothers and 68.4% of fathers born outside of the US.	Center for Epidemiologic Studies Depression Scale (CESD); Beck Anxiety Inventory (BAI).	Depression; Anxiety.	Multidimensional Acculturative Stress Inventory (MASI).	Bivariate correlations; SEM-Path analysis;. Mediation analysis; Simple slope analysis.
Menon and Harter (2012); Peer Reviewed published article	United States	Quantitative	N=590 LatinX college students. 1st & 2nd generation immigrants. Mean Age: 20.85 (SD: 4)	The Body Esteem Scale The Body Areas Satisfaction Questionnaire (BASQ) Body Mass Index (BMI)	Body Image disturbance.	The Riverside Acculturative Stress Inventory (RASI); The Sociocultural Attitude Toward Appearance Questionnaire —Revised.	Pearsons correlations; Hierarchical regression analyses; Moderation analysis.
Miller et al. (2013); Peer Reviewed published article	United States	Quantitative	Step 1: N= 288 Asian students from the Midwest university, n=158 1st generation immigrants, n=111 2nd generation immigrants. Mean Age 20.55. Step 2: N=326 Asian students, n=116 were 1st generation, n=202 were 2nd generation immigrants. Mean Age 20.99. Step 3: N=296 Asian students, Mean age: 20.83, n=107 1st generation, n=182 2nd generation immigrants.	Mental Health Inventory (MHI).	Psychological distress over the past month.	Multidimensional Acculturative Stress Inventory (MASI) – step 2; Riverside Acculturation Stress Inventory (RASI) - step 3.	K-mean cluster analysis; ANOVA.
Mwanri and Mude (2021); Peer Reviewed published article	Australia	Qualitative	N=31 (23 interviews and 1 focus group) 1st generation immigrants from Central, Eastern, and Western Africa. Mean Age: 22, ranged 18-25.	What would you say are the main health and social issues that affect young people in your community in South Australia? (what are your views about alcohol use and mental health issue among youths in African community?) 5. Why are these issues affecting youths in your community? 6. How are these issues different from when youths were in Africa? There have been cases in recent times some young people took their own lives in the African community; why do you think young people in the African community take their own lives?	Alcohol misuse & Drinking beliefs. Major Depression.	Tell me about your community in South Australia (where you have come from, social life and social networks and support etc). How has it been like for you here in Australia? Challenges /successes? What problems / challenges /issues do you think young African people face in Australia?	Qualitative approach described by Ritchie and Spencer (2002).
Nair (2017); Dissertation	United States	Quantitative	N= 220 South-East-Asian college students. 1st and 2nd generation immigrants who attended high school in the United States. Mean Age: 22.54 (SD: 3.34), Age range 18-31	The Short Depression-Happiness Scale (SDHS).	Depressed mood; happiness (mood fluctuations).	Pressure to Acculturate Scale; The Societal, Attitudinal, Familial, and Environmental Acculturative Stress Scale – 16 items (SAFE-16).	Chi square tests; ANOVA; Bivariate correlations; Multiple linear regression analysis; Hierarchical linear regression analysis.
Nguyen (2012); Dissertation	United States	Qualitative	N=6, 2nd generation Vietnamese-American women aged between 19-21.	Interview about depression and anxiety	Depression; Suicidal ideation.	Interview about acculturative stress.	Phenomenological analysis.
Oshri et al. (2014); Peer Reviewed published article	United States	Longitudinal	N=302 1st generation immigrant, Hispanic adolescents. Mean age at baseline: 14.51 (SD: 0.87). Followed up at 6, 12, 18, 24 and 30 months.	Brief Comprehensive Effects of Alcohol Scale (B-CEOA);Monitoring the Future Survey (ETOH Use); Erickson Psychosocial Stage Inventory (EPSI).	Alcohol Misuse; Alcohol expectancies; Identity coherence and confusion.	Bicultural Stress Scale administered at the first assessment (T1).	Bivariate correlations; Bivariate latent growth curve modelling.
Pagliorola (2015); Dissertation	United States	Quantitative	N=185 women (20% 1^st^ generation and 60% were 2^nd^ generation Latina or Hispanic immigrants). Age range 18-23.	The Body Shape Questionnaire, BMI	Body image & eating disorder pathology.	The Societal, Attitudinal, Familial, and Environmental Acculturative Stress Scale - 24 items (SAFE-24).	Pearson’s correlations; Linear regression analysis; Mediation analysis.
Piña-Watson et al. (2013); Peer Reviewed published article	United States	Quantitative	N=191 Mexican high school students. 58% 1st and 2nd generation immigrant. Mean age: 16.66 (SD: 1.38).	Rosenberg Self-Esteem Scale;Satisfaction with Life Scale.	Self-esteem; Life satisfaction.	Bicultural Stressors Scale.	ANOVA; Bivariate correlations; Hierarchical multiple regression analysis.
Pittman et al. (2017); Peer Reviewed published article	United States	Quantitative	N= 148, 2^nd^ generation immigrants from Africa or the Caribbean. Mean Age: 19.83 (SD: 1.54)	The Alcohol Use Disorders Identification Test (AUDIT); The Perceived Stress Scale.	Alcohol misuse; General life stress.	The Index of Race Related Stress–Brief form (IRRS-B); The Social, Attitudinal, Familial, and Environmental Acculturative Stress Scale – 24 items (SAFE-24).	Spearman’s correlations; Hierarchical multiple regression.
Polanco-Roman and Miranda (2013); Peer Reviewed published article	United States	Longitudinal	N=143. Mean age: 18.6. Age range: 18 to 25, 41% 1st generation immigrants. Nativity was assessed.	The Patient Health Questionnaire–9 (PHQ-9); The Beck Hopelessness Scale (BHS); Beck Scale for Suicidal Ideation (BSS); One question about lifetime prevalence of suicide attempts	Depression; Suicidal ideation, attempts & hopelessness.	Social, Attitudinal, Familial, and Environmental Acculturative Stress Scale (SAFE).	ANOVA; Bivariate correlations; Hierarchical linear regression; Mediation analysis.
Ponciano et al. (2020); Peer Reviewed published article	United States	Quantitative	N=748 LatinX students, n=406 from white colleges and n=342 from Hispanic Specialised Colleges. Mean Age: 19.6 (SD:1.53). 1st and 2nd generation immigrants.	Center for Epidemiologic Studies Depression Scale (CES-D)	Depression	The Multidimensional Acculturative Stress Inventory (MASI); The Perceived Discrimination Subscale from the Scale of Ethnic Experience (SEE)	A multi group path analysis; Mediation model analysis for acculturative stress.
Pulgar Guzman (2021); Dissertation	United States	Quantitative	N= 150 Hispanic young adults, 1st generation immigrants. Mean Age: 22.17 (SD: 2.22), Age range: 18-25.	Paykel Questionnaire The Patient Health Questionnaire-9 (PHQ-9)	Depression; Suicidal ideation.	Social, Attitudinal, Familial, and Environmental Acculturative Stress Scale (SAFE)	Hierarchical linear regression; Hierarchical logistic regression.
Rivera et al. (2015); Peer Reviewed published article	United States	Quantitative	N=1,100 LatinX youth. 79% of fathers and 75% of mothers were foreign born. Age range: 18–25; Mean Age: 19.71 (SD = 1.7)	Post-Traumatic Symptoms Scale - Self Report (PSS-SR). Youth Risk Behaviour Surveillance Survey. Experiences of Physical Maltreatment.	Sexual risk taking as maladaptive coping; Family Violence exposure; PTSD.	Two subscales from the Multidimensional Acculturative Stress Inventory (MASI); The seven-item Pressure to Acculturate subscale; The four-item Pressure Against Acculturation subscale.	Bivariate correlations; Simple mediation modelling; Ordinary least squares regression analysis;
Rogers-Sirin (2013); Peer Reviewed published article	United States	Quantitative	N=149 1st generation immigrant. Age range: 18-29, Mean age: 20 (SD: 2.7).	Brief Symptoms Inventory; Inventory of Attitudes towards seeking Mental Health Services (IASMHS).	Somatization; OCD; Depression; Anxiety; Psychosis; General psychological distress; Professional help seeking.	Social, Attitudinal, Familial, and Environmental Acculturative Stress Scale - Short Form (SAFE-Short).	ANOVA; Moderation analysis; Moderated mediation analysis.
Shin et al. (2021); Peer Reviewed published article	South Korea	Quantitative	N=206 adolescents from immigrant families, from eight cities, from 16 regular and three multicultural schools. Mean age: 15.81 (SD: 1.71). Non refugee 1st & 2nd generation immigrant.	Positive emotion, Engagement, Relationships, Meaning, and Accomplishment (PERMA); Youth Health Behavior Web-based Survey.	Psychological Wellbeing;	Modified version of Acculturative Stress Scale for adolescents in multicultural families.	Structural equation modelling;
Sirin et al., (2013a, 2013b); Peer Reviewed published article	United States	Longitudinal	N=286 1st and 2nd generation immigrants. Mean Age: 16.23 (SD: 0.72).	Youth Self Report (YSR) Internalising sub-scale.	Depression; Anxiety; Somatization	Social, Attitudinal, Familial, and Environmental Acculturative Stress Scale - Short Form (SAFE-Short).	Growth curve modelling; Hierarchical linear modelling (between subjects and within subjects’ measures).
Sirin et al., (2013a, 2013b); Peer Reviewed published article	United States	Longitudinal	N=332 1st and 2nd generation immigrant. Mean Age: 16.2 (SD: 1.19)	Internalizing subscale of the Youth Self-Report (YSR)	Anxiety; Depression; Somatization.	Social, Attitudinal, Familial, and Environmental Acculturative Stress Scale - Short Form (SAFE-Short).	Hierarchical linear modelling; Repeated measures ANOVA; Unconditional Growth modelling.
Stuart and Ward (2018); Peer Reviewed published article	New Zealand	Quantitative	N=155. 77% Muslim youth. 1st and 2nd generation immigrants. 23% of refugee background.. Mean Age: 20 (SD: 3.6). Age range 16-27.	10-item scale including both life satisfaction and meaning in life; A modified 12-item version of the Zung Self-rating Depression Scale.	Major Depression; Depressed mood over the past several days; Hedonic psychological wellbeing.	Modified General Perceived Discrimination Scale; Modified Cultural Readjustment Rating Scale.	Correlations; Hierarchical regression modelling.
Stuart et al. (2016); Peer Reviewed published article	New Zealand and United Kingdom	Quantitative	N= 142 from the UK, N=155 from New Zealand. 1st and 2nd generation immigrants of Muslim faith from diverse race / ethnicity including Asian, Middle Eastern, African and mixed-race. UK Mean age: 21, NZ mean age: 20. Age range 16–25.	Five items of the Diener, Emmons, Larsen, and Griffin (1985) life satisfaction scale; Behavioural problems were assessed with an 8-item scale measuring deviant behaviors, developed for the International Comparative Study of Ethno-Cultural Youth project (Bendixen & Olweus, 1999, cited in Berry et al., 2006).	Psychological wellbeing; Behavioural problems including common deviancy; life satisfaction.	Cultural Readjustment Rating Scale; Nineteen of the original 33 items were chosen based on their applicability to Muslim emerging adults.	ANOVA; Multigroup confirmatory factor analysis; Multigroup regression modelling.
Tineo et al. (2021); Peer Reviewed published article	United States	Quantitative	N= 273 Muslim college students. Mean age: 21.71 (SD: 4.53). 31.2% were 1st generation and 30.2% were 2nd generation immigrants.	Patient Health Questionnaire-8 (PHQ-8); The Generalized Anxiety Disorder-7 (GAD-7).	Depression; Anxiety.	Perceived Religious Discrimination Scale (PRDS); Social, Attitudinal, and Environmental Acculturative Stress Scale, Short Form (SAFE-Short).	Pearson's correlations; Simple mediation analysis; Moderated mediation analysis.
Tummala-Narra et al. (2016); Peer Reviewed published article	United States	Qualitative	N=16, 9 girls and 7 boys from India (n=9), Bangladesh (n=3), Pakistan (n =2), Afghanistan (n =1), and Burma (n =1). 1st generation immigrants. Mean Age16.5 (SD: 1.32), Age range: 14-18.	Probes for emotional states throughout the interview	Psychological wellbeing.	1. Tell me about what it is like for you to be a South Asian person in the United States? 2. What are some positive things about growing up as a South Asian in the U.S.? 3. What have been some hard things or difficult things about growing up as a South Asian in the U.S.? Probed for stress reactions	Conventional content analysis.
Wagaman et al. (2022); Peer Reviewed published article	United States	Quantitative	N = 462 immigrant youth with n=363 born in the USA with both parents born overseas; 32 born in the US with 1 parent foreign born and 99 were foreign born. Mean Age: 20.13 (SD 4.76).	The Coronavirus Anxiety Scale (CAS); The Identity Distress Survey (IDS).	Covid Anxiety; Identity distress.	The Social, Attitudinal, Familial, and Environmental Acculturative Stress Scale (SAFE)	ANOVA, LSD Comparison test; Bivariate correlations; Chi square test; Multiple regression analysis.
Ward et al. (2021); Peer Reviewed published article	United States	Short-term Repeated measures at Day 1 and day 12 - a 12-day diary study.	N=873 Hispanic university students. Mean age: 21 (SD: 2.8). 35% born overseas and 65% born in the USA of immigrant families.	Subtypes of Antisocial Behavior questionnaire (STAB); Center for Epidemiologic Studies Depression Scale (CES-D); Adapted version of the Beck Anxiety Inventory	Behavioural problems (aggression and defiance); depression; anxiety	Bicultural Stress Scale	Bivariate correlations; Structural equation modelling.
Wasserman et al. (2021); Peer Reviewed published article	United States	Quantitative	N= 306 Latinx adolescents and their caregivers from Nebraska. Most identified as Mexican (79.9%). 1st and 2nd 67.1% generation immigrants. Mean age: 15.50.	Center for Epidemiological Studies-Depression Scale (CES-D); Shortened DASS21	Depression; Anxiety	Multidimensional Acculturative Stress Inventory (MASI) "Pressure to Acculturate" subscale; "Pressure against Acculturation" subscale.	Bivariate correlations; Multivariate regression analyses. Moderation analysis; Simple slopes test.
Wong et al. (2017); Peer Reviewed published article	United States	Quantitative	N=306 immigrant students. Hispanic students - 31.4% 1st generation immigrant, 68.6% 2nd generation immigrants. Mean age: 21.55 (SD: 3.22); Asian students 43.8% 1st generation and 56.2% 2nd generation immigrants, mean age: 21.72 (SD: 4.78).	The Brief Symptom Inventory (BSI); Perceived Stress Scale (PSS).	Psychological distress in the last 7 days including anxious, depressed and somatising experiences; General stress.	The Social, Attitudinal, Familial, and Environmental Acculturative Stress Scale (SAFE).	Bivariate correlations; Regression analysis; Hierarchical regression analysis; Simple slope test; Path analysis.
Wang et al. (2022); Peer Reviewed published article	United States	Quantitative	N=477 Asian college students. 1st & 2nd generation. Mean Age: 20.35 (SD: 1.72),	Three-Factor Eating Questionnaire-R18; The Center for Epidemiological Studies–Depression Scale Short Form (CES-D-10).	Eating pathology; Binge eating and disinhibition; Depression.	The Riverside Acculturative Stress Inventory (RASI); The Perceived Stress Scale-14 (PSS-14)	Bivariate Pearson's correlations. Independent t-tests; Two ordinary least squares hierarchical regression analyses; Moderation analysis.
Warren and Rios (2013); Peer Reviewed published article	United States	Quantitative	N=100 male Hispanic college students. 1st & 2nd generation immigrants. Mean Age: 21 (SD: 5.47)	The Muscle Appearance Satisfaction Scale; The Sociocultural Attitudes Toward Appearance Questionnaire—3 (SATAQ-3); The Comparison to Models Survey (CMS).	Male body image disturbance	The Societal, Attitudinal, Familial, and Environmental Acculturative Stress Scale – 24 items (SAFE - 24).	Bivariate correlations; Path analysis; Mediation analysis
Zeiders et al. (2015); Peer Reviewed published article	United States	Longitudinal	N = 204 Mexican adolescent pregnant, women in their third trimester of pregnancy. Age range: 15-18. Mean Age: 16.8 (SD: 1.0). 1st and 2nd generation immigrants. Evaluated at Wave 1 (3rd trimester), wave 2,10 months later, wave 3, 24 months, wave 4, 36 months postpartum.	The Center for Epidemiologic Studies Depression Scale (CES-D)	Depression	The Multidimensional Acculturative Stress Inventory (MASI).	Multilevel growth modelling; Simple slopes test.

### Significant Relationships Between Acculturative Stress and Mental Health Problems in Immigrant Youth

Nine studies evaluated the relationship between acculturative stress and common mental health problems among immigrant youth, and this included three longitudinal studies conducted in the United States. Acculturative stress was associated with symptoms of major depression, anxiety disorders and somatic complaints over time, and these mental health problems worsened as acculturative stress increased (Polanco-Roman & Miranda, 2013; Sirin et al., 2013a, 2013b; Sirin et al., 2013a, 2013b). Acculturative stress could place immigrant youth at higher risk of developing major depression and anxiety disorders than general life stress (Katsiaficas et al., 2013; Polanco-Roman & Miranda, 2013). Polanco-Roman and Miranda (2013) also identified that high levels of acculturative stress predicted suicidal ideation after two-to-three years, by exacerbating the experience of hopelessness. Alamilla et al. (2020) found that acculturative stress predicted problematic alcohol use in immigrant young adults. Acculturative stress was also found to be related to poorer psychological wellbeing and predicted identity distress in immigrant youth (Wagaman et al., 2022). Jeon (2022) found that acculturative stress was negatively related to self-esteem among multicultural youth in South Korea.

### Significant Relationships Between Acculturative Stress and Mental Health Problems in Youth from Immigrant Minorities

#### Hispanic- and LatinX- origin Immigrant Youth Residing in the United States

Eleven studies found a positive relationship between acculturative stress, major depression and anxiety disorders in Hispanic- and LatinX-youth (Badiee & Andrade, 2019; Cano et al., 2014; Castillo et al., 2015a, 2015b; Cheng, 2022; Corona et al., 2017; Leffler, 2015; Maiya et al., 2021; Ponciano et al., 2020; Pulgar Guzman, 2021; Ward et al., 2021; Zeiders et al., 2015). Ward et al. (2021) asked Hispanic-youth to record the psychological impact of acculturative stressors across an eleven-day cycle, and found that acculturative stress could affect the emergence of symptoms of anxiety and depression within a short period of time (Ward et al., 2021). Higher levels of acculturative stress also strongly predicted lower self-esteem (Hale & Kuperminc, 2021; Piña-Watson et al., 2013). Acculturative stress was predictive of body image disturbance in male and female Hispanic youth (Menon & Harter, 2012; Warren & Rios, 2013). Among Latina female youth, greater acculturative stress was predictive of anorexia nervosa and bulimia nervosa, and related to greater media pressure to be thin, thin ideal internalisation, body shame and appearance anxiety (Claudat, 2015; Claudat et al., 2016; Menon & Harter, 2012; Pagliorola, 2015).

Ehlers et al. (2016) identified that high levels of acculturative stress along with post-migration trauma exposure and symptoms of posttraumatic stress, were predictive of alcohol misuse among emerging adults from Mexico. Oshri et al. (2014) also found that higher levels of acculturative stress were positively related to identity distress which increased the risk of alcohol misuse in Hispanic adolescents. Acculturative stress combined with posttraumatic stress disorder, trauma exposure and exposure to family violence, worsened mental health and encouraged sexual risk-taking behaviour in LatinX youth (Rivera et al., 2015). Ward et al. (2021) found that acculturative stress had a strong positive relationship with aggression and defiance in Hispanic youth.

#### Asian-origin Immigrant Youth Residing in the United States

Among Asian-origin youth, acculturative stress was related to major depression, anxiety disorders, obsessive compulsive disorder, general psychological distress, and greater acculturative stress was associated with greater severity of symptoms (Chen, 2017; Gersick, 2015). Nguyen (2012) conducted a qualitative study of six, second generation Vietnamese female youth, and two participants reported chronically high levels of acculturative stress and symptoms of depression that included suicidal ideation (Nguyen, 2012). Among South-Asian youth, high levels of acculturative stress predicted depressed mood, and South-Asian youth who described experiences of acculturative stress, also reported experiencing identity distress, difficulties with relationships, and ongoing psychological distress (Nair, 2017; Tummala-Narra et al., 2016). Among Asian female youth, acculturative stress was a predictor of eating pathology such as anorexia nervosa and bulimia nervosa (Claudat et al., 2016).Wang et al. (2022) examined the relationship between acculturative stress and binge eating in Asian-origin youth, and acculturative stress predicted binge eating behaviour irrespective of body weight.

#### Muslim- and Middle-Eastern-origin Immigrant Youth Residing in the United States, New Zealand, United Kingdom and Australia

Tineo et al. (2021) studied Muslim-youth living in the United States and found that acculturative stress was positively associated with major depression and generalised anxiety disorder. Assar (2015) also found that among Middle-Eastern youth in the United States, acculturative stress predicted self-esteem which predicted major depression. Higher levels of acculturative stress were related to higher levels of psychological distress, lower self-esteem and poorer social competence in mainly Middle-Eastern, Muslim adolescents in the United States, and acculturative stress was significantly related to depressed mood and lower life satisfaction in Muslim- youth residing in New Zealand (Goforth et al., 2014; Ibrahim-Joudeh, 2014; Stuart & Ward, 2018). For Muslim youth in New Zealand and the United Kingdom, greater acculturative stress increased the likelihood of deviant behaviour, such as vandalising public property (Stuart et al., 2016).

#### African-origin Immigrant Youth and Refugee Youth Residing in the United States, Australia & South Korea

Alemi et al. (2021) interviewed Somalian refugees in the United States, who reported how acculturative stress, ethnic discrimination and parental invalidation exacerbated experiences of depression and posttraumatic stress disorder (PTSD). Pittman et al. (2017) studied African- and Caribbean- youth in the United States and found acculturative stress to be positively related to problematic alcohol use above general stress and race related stress. Acculturative stressors including family separation, arriving unaccompanied, educational disruptions, low English proficiency, poverty, high expectations from country-of-origin communities and lack of country-of-settlement supports, all which contributed the breakdown in relationships and reputation, resulting in very high levels of acculturative stress, symptoms of depression and encouraging alcohol misuse as a coping strategy (Mwanri & Mude, 2021). Among refugee youth living in South Korea, acculturative stress was predictive of anxiety, depression and posttraumatic stress disorder, more so than pre-settlement trauma exposure (Kim et al., 2015).

### Factors That Influence the Relationship between Acculturative Stress and Mental Health Among Youth

#### Acculturation

Settlement-country language proficiency, multilingualism and greater time in settlement, were identified as strengths among immigrant youth with lower levels of acculturative stress and better psychological adjustment (Bhowmik, 2021; Goforth et al., 2014; Ibrahim-Joudeh, 2014). Furthermore, culutral competency and identification with the settlement culture affected the relationship between acculutrative stress and mental health problems among immigrant minority youth. Among LatinX-youth with low levels of cultural competency for the United States, acculturative stress caused from family acculturative stressors predicted symptoms of anxiety, and school-based acculturative stressors predicted depression (Leffler, 2015). Castillo et al., (2015a, 2015b) found that among Hispanic-male youth with greater cultural competency for the United States, depression was related to acculturative stress caused by the pressure to retain heritage culture and language. Nair (2017) found that among Asian-origin youth, lower cultural identification with United States was associated with higher levels of acculturative stress and poorer psychological wellbeing.

Heritage-cultural identity also influenced the relationship between acculturative stress and mental health among immigrant minority youth. Among Asian-orign youth in the United States, lower heritage-culture identification was associated with reduced capacity to cope with acculturative stress (Miller et al., 2013; Nair, 2017; Tummala-Narra et al., 2016). Similarly, Piña-Watson et al. (2013) found that greater heritage-cultural identification was protective of psychological wellbeing when acculturative stress was high among Mexican-youth. Jankowski et al. (2020) found a strong relationship between acculturative stress and alcohol misuse when religious identity and practice was high, cultural identification with the United States was low, and heritage-cultural identity was high among Asian- and Hispanic- youth. Among LatinX youth, greater cultural incongruity between heritage-culture and the culture of the United States, strengthened the relationship between acculturative stress and major depression, irrespective of the length of time in the United States (Cano et al., 2014; Ponciano et al., 2020).

#### Social Support

Social support was identified as a protective factor that weakened the relationship between acculturative stress and major depression and anxiety, especially at the ages of 15 to 16 (Katsiaficas et al., 2013; Sirin et al., 2013a, 2013b). Social support was identified as a factor that influenced acculturative stress and its relationship to psychological wellbeing and psychological distress among immigrant youth in Hong Kong/China, South Korea and the United States (Bhowmik, 2021; Miller et al., 2013; Shin et al., 2021; Tummala-Narra et al., 2016; Wagaman et al., 2022). Menon and Harter (2012) identified social support as being protective against the effects of acculturative stress on eating pathology in LatinX-youth by being able to lower body-image disturbance in the presence of high levels of acculturative stress.

#### Family functioning

Among Hispanic / LatinX youth in the United States, maternal warmth, family bonding and core Hispanic family values were found to weaken the relationships between acculturative stress, psychological distress and depression, however, low levels of family support strengthened the relationship between acculturative stress and depression (Corona et al., 2017; Hale & Kuperminc, 2021; Leffler, 2015; Zeiders et al., 2015). Furthermore, Chen (2017) identified that among Asian male youth and only-children in the United States, adultification towards their families, filial piety and parental immigration, increased acculturative stress and strengthened its relationship with major depression and anxiety disorders. Stuart et al. (2016) identified that greater perceived family obligation was associated with higher levels of acculturative stress, which were related to behavioural problems among Middle-Eastern youth in New Zealand and the United Kingdom (Stuart et al., 2016).

#### Religious Identity and Practice

Religious identity strengthened the relationship between acculturative stress, major depression and generalised anxiety disorder among Muslim-immigrant youth in the United States, (Alemi et al., 2021; Hernandez Dubon et al., 2022; Tineo et al., 2021). Among immigrant youth with Muslim religious identification, perceived discrimination and religious stereotyping exacerbated the impact of acculturative stress on hopelessness, major depression and anxiety disorders (Alemi et al., 2021; Assar, 2015; Tineo et al., 2021). However, religious identity, practice and support was also reported as comforting, and was negatively associated with acculturative stress among youth of Muslim identity in the United States and New Zealand (Alemi et al., 2021; Goforth et al., 2014; Stuart & Ward, 2018).

#### Immigrant Generation Status

This review included studies of youth who had immigrated themselves (first-generation immigrant) or, who had been born in their settlement country and had parents who had immigrated (second-generation immigrant). Five studies compared generation status (first- and second-generation) among immigrant youth in the United States. Significant relationships between acculturative stress and depression and psychological wellbeing were identified irrespective of generation status (Katsiaficas et al., 2013; Wagaman et al., 2022). Two longitudinal studies also indicated that generation status did not affect the relationship between acculturative stress and depression, anxiety and somatisation across time (Sirin et al., 2013a, 2013b; Sirin et al., 2013a, 2013b). However, Alamilla et al. (2020) found a significant relationship between acculturative stress and problematic alcohol use only in second generation immigrant youth born in the United States. Among studies of Hispanic / LatinX youth in the United States, generation status was not significantly related to acculturative stress, or mediated or moderated the relationship between acculturative stress, major depression, anxiety disorders, body image disturbance or eating pathology (Badiee & Andrade, 2019; Castillo et al., 2015a, 2015b; Corona et al., 2017; Ehlers et al., 2016; Lee et al., 2018; Menon & Harter, 2012; Pagliorola, 2015; Piña-Watson et al., 2013; Rivera et al., 2015; Wong et al., 2017; Zeiders et al., 2015). Among Asian-origin youth in the United States, generation status did not influence the relationship between acculturative stress and eating disorders or major depression (Wang et al., 2022). Among Muslim-immigrant youth in the United States, New Zealand and the United Kingdom, generation status did not influence the relationship between acculturative stress and life satisfaction, behavioural problems or depression and anxiety (Stuart et al., 2016; Tineo et al., 2021).

## Discussion

Among immigrants, acculturation is the ongoing psychological and behavioural responsiveness required for the continuous bicultural integration into the settlement environment (Sam & Berry, 2016). The pervasive, negative emotional discomfort that includes distress and worry which results from the accumulation of acculturative stressors is termed ‘acculturative stress’ (Berry & Annis, 1974; Berry et al., 1987). Acculturative stress is not culture shock or general life stress, but the accumulation of stress experienced in the face of complicated circumstances, directly resulting from the need to acculturate and live a bicultural life (Smart et al., 2001; Williams & Berry, 1991). For youth in the stages of late adolescence and early adulthood, being an immigrant needing to acculturate, has been identified as a risk factor contributing to serious mental health problems in countries that welcome immigrants (Abraham & Sher, 2021; Kim et al., 2019). This scoping review focused on recent research on acculturative stress and its relationship with mental health problems among immigrant youth. This review has confirmed that acculturative stress continues to be a highly relevant problem that is significantly related to the mental health of youth. This review has noted that acculturative stress is experienced by immigrant youth, irrespective of the length of time in settlement or birthplace, and can consume emotional resources, leading to an increased risk of emerging mental health problems (Sirin et al., 2013a, 2013b; Wagaman et al., 2022).

Berry et al. (1987) identified four key components to the acculturation process that determine the experience of acculturative stress. This includes the context of the settlement environment; the cultural or ethnic group, their history of migration and attitudes to acculturation; the ability of the individual to manage a bicultural identity and environment; and socioeconomic factors (Berry & Annis, 1974; Berry et al., 1987; Hovey, 1999). Therefore, it was important to expand this review to include studies focused on youth from immigrant minority groups. These studies provided specific examples and models of assessing acculturative stress and mental health problems, among these youth. These studies were conducted in a manner that provided a tailored understanding of the experience of acculturative stress and stressors, specifically for the context of the settlement environment, the immigrant group, the developmental stage of late adolescence and emerging adulthood and included youth of first- and second-generation immigrant status who experienced acculturative stress. Studies conducted with Hispanic / LatinX - origin youth, Asian-origin youth, African-origin youth, Middle-Eastern-origin youth and Muslim immigrant youth, reinforced the role of acculturative stress in emerging mental health problems such as; major depression, anxiety disorders, behavioural disturbance, eating disorders and poor psychological wellbeing among immigrant minority youth. In some studies, minority stress resulting from the disempowered position of minority groups, including religious minority groups, was considered part of the acculturative stress experience (Corona et al., 2017; Jeon, 2022; Tineo et al., 2021). However, in studies that included Hispanic and LatinX-origin youth and African or Caribbean-origin in the United States, experiences of minority stress were considered a separate factor affecting the mental health of youth (Pittman et al., 2017; Ponciano et al., 2020). Acculturative stress among Hispanic and LatinX-origin youth residing in the United States, was reported to mainly result from pressures to acculturate and pressures against acculturation (Ponciano et al., 2020; Wasserman et al., 2021). This is an example of how sources of acculturative stress can differ between groups of immigrant youth, but nevertheless the experience of acculturative stress continues to play a significant role in the emergence of mental health problems across different groups albeit in different ways.

The majority of studies that fulfilled inclusion criteria were conducted in the United States. This allowed an in-depth understanding of the experiences of immigrant youth of this settlement country and context, where 26% of the total population under 18 years of age are currently the children of immigrants (Batalova, 2024). This review also included studies conducted in multicultural societies such as Australia, New Zealand, the United Kingdom, China and South Korea and included youth from refugee backgrounds. Although these were only a total of seven studies, they provide encouraging evidence that acculturative stress is related to the mental health and psychological wellbeing of youth across settlement countries, and that this is a relevant and important relationship affecting the mental health of immigrant youth including youth of refugee background (Mwanri & Mude, 2021; Williams & Berry, 1991; Yako & Biswas, 2014).

### Recommendations for Researchers

The high number of studies conducted in the United States provided substantial understanding regarding the relationship between acculturative stressors, stress and emerging mental health problems that highlighted the impact of the settlement country (Berry et al., 1987). Future research should steer towards investigating the relationship between acculturative stress and problems with mental health in immigrant youth within other countries that offer the long-term settlement of immigrants. Research efforts could include a variety of individual studies on immigrant youth of mixed background, focused studies on groups of immigrant minority youth, studies of refugee youth and studies of youth from religious minorities. A mixture of research methods should also be applied to capture the complexity and diversity of acculturative stressors that result in acculturative stress and impact the mental health of youth in a manner that advocates for their needs.

This scoping review included a small number of qualitative studies that examined the complexity and accumulative effects of acculturative stressors especially in immigrant minority youth. The accounts of these experiences provided primary evidence identifying acculturative stressors and stress, and their accumulative effect on the mental health of participants (Bhowmik, 2021; Nguyen, 2012). Qualitative research methods would prioritise the perspective of participants in describing experiences of acculturation specific to the context of youth. Future research should steer towards including qualitative methods in studies of acculturative stress and mental health.

The mental health of youth from refugee backgrounds has been shown to be severely affected by the impact of humanitarian circumstances and acculturation experiences (Halcon et al., 2004; Kim et al., 2015). Additional focus should be given to youth with a proximal history of migration due to humanitarian circumstances. This review identified only a small number of studies investigating acculturative stress and mental health problems in refugee youth that indicated severe distress, and a complex landscape of acculturative stressors (Alemi et al., 2021; Mwanri & Mude, 2021). Investigating the experience of acculturative stress and its relationship to the mental health of refugee youth should be a priority for future research specialising in the needs of refugee youth (Williams & Berry, 1991).

Substantial effort was taken to choose a definition of ‘youth’ that would be understood in the context of international migration and relevant in multiple settings. The United Nations Department of Social and Economic Affairs (2013) defined youth as *“a period of transition from the dependence of childhood to adulthood’s independence*” and this definition supported the focus of this review. However, identifying studies that captured this age group that included late adolescence and emerging adulthood proved challenging because age groups that define youth can vary greatly (UNDESA 2013). The majority of studies in this review focused on emerging adulthood and only ten studies focused on acculturative stress and mental health in late adolescence. Future studies that aim to address such as global issue may consider research focused on youth aged 15-24, capturing the experiences of youth in the stages of later adolescence and emerging adulthood.

The most common instruments for assessing acculturative stress among these studies was the Social, Attitudinal, Familial and Environmental Acculturative Stress Scale (SAFE), followed by the Multidimensional Acculturative Stress Scale (MASI), the Riverside Acculturative Stress Inventory (RASI), and the Acculturative Stress Scale. These instruments evaluated stress responses elicited from the pressure to acculturate, pressure against acculturation, family and intergenerational conflict, communication difficulties, social isolation, cultural fit, minority stress and discrimination and intercultural relations (Miller et al., 2011; Rodriguez et al., 2002; Sandhu & Asrabadi, 1994; Suh et al., 2016). Efforts should continue in developing valid and reliable models of assessment and instruments that assess acculturative stress in immigrant youth, across settlement countries and immigrant groups so they can be used as part of supportive practice dedicated to the mental health of immigrant youth.

### Recommendations for Practitioners Supporting the Mental Health of Immigrant Youth

Acculturative stress is significantly related to the mental health of immigrant youth irrespective of the length of time in the settlement-country or birthplace and should be directly addressed when providing mental health care (Claudat et al., 2016; Hale & Kuperminc, 2021; Sirin et al., 2013a, 2013b). Acculturative stress includes symptoms such as; excessive worry, psychological distress, identity confusion, depressed mood, feelings of hopelessness and isolation, somatisation and interpersonal stress that result from acculturation (Williams & Berry, 1991; Yako & Biswas, 2014). Acculturative stress can be assessed by reviewing the effects of acculturation experiences, or using assessment tools such as those identified in this review (Nguyen, 2012; Umana-Taylor & Alfaro, 2009). When providing mental health care, practitioners should seek to alleviate acculturative stress as part of psychological treatment. This could include; enhancing social supports, developing effective social skills, encouraging religious practice, exploring family values and relationships, managing cultural incongruity and addressing cognitive appraisals about stressors, future goals and self-esteem (Bhowmik, 2021; Cheng, 2022; Jeon, 2022; Kim et al., 2015; Rivera et al., 2015). Using a strengths-based approach to coping with acculturative stress that encourages self-reflection, positive self-appraisal, differentiation-of-self, ethnocultural empathy and an alternating cultural identity style, have been recommended as psychological approaches that alleviate acculturative stress (Chen, 2017; Gersick, 2015; Piña-Watson et al., 2013; Wei et al., 2016).

It is critical that practitioners are culturally competent and attuned to the experiences of immigrant youth (Corona et al., 2017; Katsiaficas et al., 2013; Wagaman et al., 2022). Practitioners providing mental health care to immigrant youth should be developmentally informed, and considerate of cultural safety. Professional development activities may include; consultation or collaboration with community advisers about the acculturation challenges of local immigrant communities; an understanding of the historical and current context of immigration in the settlement country; and knowledge about international migration patterns (Alemi et al., 2021; Mwanri & Mude, 2021; Tineo et al., 2021).

### Recommendations for Policies Supporting Immigrant Youth

Acculturative stress results from the accumulation of acculturative stressors. Supportive policies focused on immigrant youth, should focus on alleviating the experience of acculturative stress by supporting immigrant youth in educational, vocational and community settings. The period between late adolescence and emerging adulthood is a critical time for educational and vocational achievement and school engagement impacts acculturation and acculturative stress (Alemi et al., 2021; Ponciano et al., 2020). Academic settings should make substantial efforts to reduce discrimination and ethnic and religious stereotyping, and increase opportunities for supporting the heritage or ethnic cultural identities of their immigrant students, as well as their bicultural integration (Leffler, 2015; Nair, 2017; Piña-Watson et al., 2013; Stuart & Ward, 2018). Additional acculturation challenges that were found to mediate the relationship between acculturative stress and common mental health problems in immigrant youth included; maintaining bicultural communication skills, cultural incongruity with settlement culture and cultural competency for the settlement country (Bhowmik, 2021; Cano et al., 2014; Jankowski et al., 2020; Miller et al., 2013). Policies supporting youth in education and community settings, should consider the value of bilingual communication skills and support multilingual language development, alleviate deficits in cultural competency for the settlement culture, especially in groups of recently settled immigrant youth, and where there is evidence of cultural incongruity (Castillo et al., 2015a, 2015b; Katsiaficas et al., 2013; Miller et al., 2013).

Academic and vocational support should be diverse and easily accessible for immigrant youth (Cano et al., 2014; Ponciano et al., 2020; Wasserman et al., 2021). Supportive services should also be tailored to avoid stigma and enhance help-seeking in the community and include religious and cultural settings (Alamilla et al., 2020; Alemi et al., 2021; Katsiaficas et al., 2013; Wasserman et al., 2021). Furthermore, social support from family and peers loosened the relationship between acculturative stress and common mental health problems, and both emotional and practical support was valued among immigrant youth (Katsiaficas et al., 2013; Menon & Harter, 2012; Sirin et al., 2013a, 2013b). Policy makers should consider providing greater opportunities for leisure activities, socialisation and networking for immigrant youth, (Maiya et al., 2021; Shin et al., 2021).

Addressing family factors has developmental and acculturative benefits and should be considered when attempting to develop supportive environments related to youth (NASEM 2019). Family functioning both mediated and moderated the relationship between acculturative stress and mental health problems across various groups of immigrant youth (Corona et al., 2017; Stuart et al., 2016; Wasserman et al., 2021). Supporting the acculturation of families should be considered as part of policies providing supportive environments to immigrant youth (Stuart et al., 2016). Immigrant youth can be responsible for the welfare and adaptation of their families, which is a significant stressor (Chen, 2017; Stuart et al., 2016). Furthermore, core family values can differ between settlement and heritage cultures (Corona et al., 2017; Leffler, 2015). Supportive policies that focus on immigrant youth should also include policies related to their families and consider family relationships, attitudes and responsibilities that may contribute to acculturative stress (Cheng, 2022).

## Strengths and Limitations

This review was unique in that is focused on exploring research published between 2012 and 2022, related to acculturative stress and mental health for immigrant youth mainly aged between 15-24. This review had several strengths including; a rigorous search strategy that accessed a wide range of databases; the lack of geographical limits; the inclusion of a range of study designs; and the inclusion of studies of immigrant minority groups. This allowed the identification of a wide breadth of evidence for this review which was truly exploratory but also focused on understanding the experience of immigrant youth. The authors also used evidence-based processes such as JBI methodology and PRISMA-ScR guidelines to conduct this review (Page et al., 2021; Tricco et al., 2018). However, this review did not include a quality assessment of the included studies, and data extraction from the included studies was conducted only by the primary author (Peters et al., 2017). For these reasons, the authors attempted to be cautious about the interpretation of findings and Table 2 included a summary of study methods for readers’ consideration (Peters et al., 2017). Additionally, studies were excluded from this review if an English translation was not available, limiting the representation of non-English speaking settlement countries.

## Conclusion

Rates of international migration are increasing at an accelerated rate, and immigrating is a complex experience among obstacles such as, historically high levels of global conflict and changing political and economic conditions. Growing populations of immigrant youth facing the challenges of long-term settlement continue to experience difficulties with their mental health and there is an urgent need to support the settlement and wellbeing of immigrant youth and especially of refugee youth. Acculturative stress continues to play a significant role in the emergence of serious mental health problems in recent times and should continue to be considered an integral part of policy and practice when addressing the emergence of mental problems among youth, in countries that provide long-term settlement to immigrants. Acculturative stressors are specifically tailored to the context of immigration that include immigrant group and settlement country factors, bicultural adaptation, as well as the sensitivity to stress and critical stage of development that youth find themselves in. For these reasons, learning to live an adaptive, bicultural life is complex, stressful and can affect the health status of immigrant youth. A strength of this review was its inclusion of an array of study methods, settlement countries and immigrant minority youth. These criteria have offered a variety of evidence that examined the state of research and understanding of this topic in recent times. For these reasons, future research efforts should continue to concentrate on evaluating the acculturative stressors and stress-related experiences faced by immigrant youth, and prioritise interventions that alleviate features of acculturative stress, as an essential part of developing supportive environments, and therapeutic interventions that are relevant to the current pressures faced by youth of immigrant background.

## Footnotes

1 In this study, *immigrant(s)* is the term used to describe any person or people who have lived experience of international migration, who themselves had arrived at their settlement country either through humanitarian or non-humanitarian pathways.
